# Comparative Sequence Analysis of Historic and Current Porcine Rotavirus C Strains and Their Pathogenesis in 3-Day-Old and 3-Week-Old Piglets

**DOI:** 10.3389/fmicb.2020.00780

**Published:** 2020-04-24

**Authors:** Juliet Chepngeno, Sayaka Takanashi, Annika Diaz, Husheem Michael, Francine C. Paim, Michael C. Rahe, Jeffrey R. Hayes, Courtney Baker, Douglas Marthaler, Linda J. Saif, Anastasia N. Vlasova

**Affiliations:** ^1^Food Animal Health Research Program, Ohio Agricultural Research and Development Center, Department of Veterinary Preventive Medicine, College of Food, Agricultural and Environmental Sciences, The Ohio State University, Wooster, OH, United States; ^2^Department of Developmental Medical Sciences, The University of Tokyo, Tokyo, Japan; ^3^Department of Animal Sciences, College of Food, Agricultural and Environmental Sciences, The Ohio State University, Columbus, OH, United States; ^4^Department of Veterinary Diagnostic and Production Animal Medicine, Iowa State University, Ames, IA, United States; ^5^Animal Disease Diagnostic Laboratory, The Ohio Department of Agriculture, Reynoldsburg, OH, United States; ^6^Kansas State Veterinary Diagnostic Laboratory, College of Veterinary Medicine, Kansas State University, Manhattan, KS, United States

**Keywords:** porcine rotavirus, group C, characterization, pathogenesis, United States

## Abstract

The increased prevalence of porcine group C rotavirus (PRVC) in suckling piglets and the emergence of new genetically distinct PRVC strains are concerning due to the associated significant economic losses they cause to the swine industry. We sequenced and analyzed two new PRVC strains, RV0104 (G3), and RV0143 (G6) and compared their pathogenesis with that of the historic strain Cowden (G1) in gnotobiotic (Gn) pigs. Near complete genome sequence analysis confirmed that these two strains were distinct from one another and the Cowden strain. VP1, VP2, VP6, NSP1-NSP3, and NSP5 genes were more similar between Cowden and RV0143, whereas VP3, VP7, and NSP4 shared higher nucleotide identity between Cowden and RV0104. Three-day-old and 3-week-old Gn piglets were inoculated with 10^5^ FFU/piglet of Cowden, RV0104 or RV0143, or mock. All 3-day-old piglets developed severe diarrhea, anorexia, and lethargy, with mean PRVC fecal shedding titers peaking and numerically higher in RV0104 and RV0143 piglets on post infection day (PID) 2. Histopathological examination of the small intestine revealed that the 3-day-old Cowden and RV0104 inoculated piglets were mildly affected, while significant destruction of small intestinal villi was observed in the RV0143 inoculated piglets. Consistent with the highest degree of pathological changes in the small intestines, the RV0143 inoculated piglets had numerically higher levels of serum IL-17 and IFN-α cytokines and numerically lower PRVC IgA geometric mean antibody titers. Milder pathological changes and overall higher titers of PRVC IgA antibodies were observed in 3-week-old vs. 3-day-old piglets. Additionally, diarrhea was only observed in RV0104 and RV0143 (but not Cowden) inoculated 3-week-old piglets, while levels of serum IL-10 and PRVC IgA antibodies were higher in Cowden inoculated pigs, consistent with the lack of diarrhea. Thus, we confirmed that these current, genetically heterogeneous PRVC strains possess distinct pathobiological characteristics that may contribute to the increased prevalence of PRVC diarrhea in neonatal suckling piglets.

## Introduction

Rotaviruses (RVs) infect small intestinal mature enterocytes on the tips of the villi in the small intestines causing diarrhea and destruction of these enterocytes (Estes et al., 2001). Several mechanisms of diarrhea induction by RVs have been described: malabsorption of fluids and electrolytes that occurs due to the extensive damage to small intestinal villous epithelium, activation of the enteric nervous system induced by neurological dysfunction and the resulting disruption of blood flow due to damaged enterocytes, and the action of RV non-structural protein, NSP4, an enterotoxin that increases intracellular Ca^2+^ levels further disrupting fluid homeostasis in infected and non-infected adjacent cells (Ruiz et al., 2000; Estes et al., 2001; Vlasova et al., 2017).

The rotavirus genome consists of 11 double-stranded RNA (dsRNA) segments encoding six structural proteins (VP1, VP2, VP3, VP4, VP6, and VP7) and five or six non-structural proteins (NSP1–NSP5/6), the latter depending on translated open reading frame of segment 11 (Estes and Cohen, 1989). A complete genome-based classification system comprising the RV 11 dsRNA segments was proposed by the Rotavirus Classification Working Group (Matthijnssens et al., 2008a, b; McDonald et al., 2009). The complete genome analysis of rotavirus group C (RVC) strains has been done recently and it confirmed the existence of at least 18G, 21P, 13I, 4R, 6C, 6M, 9A, 8N, 6T, 5E, and 4H genotypes for the genes VP7, VP4, VP6, VP1, VP2, VP3, NSP1, NSP2, NSP3, NSP4, and NSP5, respectively, in terrestrial mammals (Suzuki and Hesebe, 2017).

Despite the increased in demand for pork and pork products globally, the swine industry is facing significant economic losses due to the high disease burden associated with enteric pathogens. Pigs that suffer from RV diarrheal disease exhibit stunted growth, anorexia, lethargy or even mortality, resulting in significant economic losses (Saif, 1999). Porcine rotavirus group C (PRVC) was first discovered in 1980 (Saif et al., 1980) and is currently recognized as the major single cause of gastroenteritis in neonatal piglets (Marthaler et al., 2014). Recently, numerous investigators have reported on the increased prevalence of PRVC in swine worldwide (Morin et al., 1990; Martella et al., 2007, Marthaler et al., 2014, Moutelikova et al., 2014). In contrast to RVA, the lack of a robust cell culture (except Cowden G1 genotype; Saif et al., 1988) and its sporadic nature in the past have resulted in limited knowledge on RVC epidemiology, pathogenesis, and immunity. Understanding the pathogenesis of these strains will allow diagnostic tools and preventive measures to be developed, since RVC is increasingly being detected in both animals and humans and interspecies and zoonotic transmission has been confirmed (Saif et al., 1980; Rahman et al., 2005; Amimo et al., 2013; Marthaler et al., 2014; Kattoor et al., 2017; Vlasova et al., 2017).

Previously, our lab demonstrated a high prevalence of PRVC in commercial swine farms in Ohio, United States and the dominance of new PRVC genotypes G3 and G6 on these farms associated with RVC disease in neonatal piglets (Amimo et al., 2013). The same genotypes are confirmed as circulating in other parts of the United States and Canada (Marthaler et al., 2013). The reasons for the increase in the prevalence of PRVC infection and disease in neonatal piglets are not well understood. With the emergence of new PRVC genotypes of unknown pathogenicity, and the lack of effective vaccines, comparative studies are necessary to develop strategies for reliable control and prevention measures. In this study, we comparatively sequenced and evaluated the pathogenesis of the two new PRVC strains, RV0104 and RV0143, and the historic strain Cowden.

## Materials and Methods

### Viruses

RV0104 G3 and RV0143 G6 PRVC strains, originating from feces of nursing piglets in two swine farms in Ohio were diluted 1:10 in sterile Minimal Essential Medium (MEM Gibco; Life Technologies, Grand Island, NY, United States), clarified by centrifugation at 2000 × *g* for 15 min at 4°C, and filtered through 0.2 μm filter. The presence of other enteric viruses that causes diarrhea was screened using RT-PCR. RNA isolated as above was also tested for porcine RVA and porcine RVB using RT-PCR and specific primers as described in Amimo et al. (2013). Porcine epidemic diarrhea virus (PEDV) and porcine deltacoroviruses were detected using conventional PCR as described in Jung et al. (2014), Vlasova et al. (2011), and Hu et al. (2018). Furthermore, complete genome sequencing has been performed using Next generation sequencing (NGS) that also confirmed absence of other viral pathogens. To conduct the experiments, small and large intestinal contents (*SIC* and LIC) of actively infected (7–10 day-old) Gn piglets were pooled, diluted, filtered (as above), and used as viral stock inoculum as noted above for the other PRVC strains. The titers of each inoculum were determined by RT-qPCR using a standard curve that was developed in this study with RT-qPCR of 10-fold serial dilutions of synthetic genes of Cowden, RV0104 and RV0143 (RV0104 VP6 accession # MN809647.1, RV0143 VP6 accession # KC164677.1, and Cowden VP6 accession # M94157.1) and RVC diagnostic primers (Amimo et al., 2013) obtained from Integrated DNA Technologies, Inc.1710 Commercial Park Coralville, IA, United States.

These Gn pig pools were then used for sequencing and to orally inoculate Gn piglets (3-day-old and 3-week-old). Some piglets were euthanized on post inoculation day (PID) 3 to assess the intestinal pathology, while the rest were euthanized at PID10. The original virulent Cowden, G1 strain (Saif et al., 1980) was serially passaged to maintain virulence in Gn piglets 17 times.

### Next Generation Sequencing

For NGS previously extracted RNA underwent cDNA synthesis according to a random primer protocol performed using RevertAid H Minus First Strand cDNA Synthesis Kit (Thermo Scientific, Waltham, MA, United States). PCR was conducted using True-Start DNA polymerase with 10 mM dNTPs mix and 10 pmol specific primers per reaction (Thermo Scientific, Waltham, MA, United States), according to the manufacturer’s protocols. TruSeq Stranded Total RNA Library Prep Kit was used with 1 μg total RNA for the construction of libraries according to the manufacturer’s protocol. For rRNA-depleted library, rRNA was removed from 2.5 μg total RNA using Ribo-Zero rRNA Removal Kit (mixture 1:1 Human/Mouse/Rat probe and Bacteria probe), according to the manufacturer’s protocol (with probe concentration for epidemiology kit protocol). All cDNA libraries were sequenced using an Illumina HiSeq2000 (Illumina, San Diego, CA, United States), producing 101 × 7 × 101 bp paired end reads with multiplexing. Reads were trimmed using default parameters with CLC Genomics Workbench 8.5.1 (Qiagen Bioinformatics, Redwood City, CA, United States). Trimmed reads were *de novo* assembled using a word size of 64, bubble size of 100, and minimum contig length of 100. The contigs were subject to the Basic Local Alignment Search Tool (BLASTn) to identify the RVC strains sharing the highest nucleotide identity with the RV0104 and RV0143 strains. RVC sequences were deposited into GenBank with the accession numbers.

### Sanger Sequencing

Amplified structural genes RVC RV0104 VP4 was purified by QIAGEN Gel Extraction Kit (Qiagen, Hilden, Germany) as described by the manufacturer with three sets of primers (Supplementary Material). Expected size of DNA fragment was excised from the 1% agarose gel and dissolved using buffer QG (3× of gel weight) at 50°C. Once the gel dissolved, 1 gel volume of isopropanol was added, and the solution was transferred into the spin column. Columns were centrifuged for 1 min at 10,000 × *g* 4°C, flow through discarded, 500 μL of buffer QG was added, and centrifuged again at 10,000 × *g* 4°C at 1 min and flow through was discarded. 750 μL of buffer PE was added to columns, centrifuged at 10,000 × *g* 4°C for 1 min and flow through was discarded. The spin column was transferred to a clean 1.5 ml micro centrifuge tube and the DNA bound to the column was eluted using 50 μL of elution buffer by centrifugation at 10,000 × *g* 4°C for 1 min. PCR products along with corresponding forward and reverse primers (Supplementary Material) were submitted to the molecular and cellular imaging center (MCIC), OARDC, Wooster, Ohio for sanger sequencing and the sequence assembled with VP4 sequence obtained from NGS mentioned above. The GenBank accession numbers of RVC/Pig-wt/USA/RV0143/2012 are MN809633 (VP1), MN809634 (VP2), MN809635 (VP3), MN809636 (VP4), MN809637 (VP6), MN809638 (VP7), MN809639 (NSP1), MN809640 (NSP2), MN809641 (NSP3), MN809642 (NSP4), MN809643(NSP5), RVC/Pig-wt/USA/RV0104/2011 are MN809644 (VP1), MN809645 (VP2), MN809646 (VP3), MT181131(VP4), MN809647(VP6), MN809648(VP7), MN809649(NSP1), MN809650(NSP2), MN809651 (NSP3), KC164673 (NSP4), MN809652 (NSP5), and RVC Cowden (G1) M74216(VP1), FJ970917 (VP2), M74219 (VP3), M74218 (VP4), M94157(VP6), M61101(VP7), X60546 (NSP1), X65939 (NSP2), M69115(NSP3), AF093202 (NSP4), and X65938 (NSP5).

### Animals and Experimental Design

Gn piglets were derived by cesarean section and maintained under germ-free conditions as described previously (Meyer et al., 1964). Each PRVC inoculum (Cowden, RV0104, and RV0143) was diluted in MEM to 10^5^ FFU/ml. One ml of each inoculum was used to orally inoculate 3-day-old and 3-week-old Gn piglets immediately after feeding 3 ml of 100 mm sodium bicarbonate to reduce stomach acidity. Piglets were assigned to one of eight different groups: 3-day-old; RV0104 (*n* = 7), Cowden (*n* = 5), RV0143 (*n* = 7), and mock (*n* = 4) and 3-week-old; RV0104 (*n* = 7), Cowden (*n* = 5), RV0143 (*n* = 7), and mock (*n* = 3; Table 1). Mock piglets were inoculated with 1 ml of MEM. Pigs were examined daily for diarrhea and their fecal scores noted as follows: 0 –normal = solid; 1- pasty; 2- semi-liquid; and 3, watery diarrhea.

**TABLE 1:** Summary of porcine RVC diarrhea and fecal virus shedding in Gn piglets after PRVC inoculation (PID1 to PID10).

		Virus shedding				Diarrhea*			
			
Experiment Groups	*N*	% shed	Mean days to onset of shedding	Mean duration days	Mean peak titer shed (GE copy/ml)	% with diarrhea	Mean days to onset of diarrhea	Mean duration days^†^	Median cumulative fecal score^†@^
**3-day-old**									
Cowden	5	100	<1	9	1.32 × 10^6^	100	2	7.3	18.3^B^
RV0104	7	100	<1	9	3.78 × 10^7^	100	2	5.75	14.25^A^
RV0143	7	100	<1	9	2.28 × 10^7^	100	1.75	6.5	16^B^
Mock	4	0	N/A	N/A	N/A	0	N/A	N/A	N/A
**3-week-old**									
Cowden	5	100	3.33^A^	6.67^A^	1.36 × 10^10A^	0	N/A	0	5.4^A^
RV0104	7	100	2.87^A^	7.17^A^	4.9 × 10^9A^	100	2.75^A^	2.75^A^	10.5^B^
RV0143	7	100	1.75^B^	8.25^B^	5.33 × 10^11B^	100	3.6^B^	3.7^B^	10.8^B^
Mock	3	0	N/A	N/A	N/A	0	N/A	N/A	N/A

*Pigs with fecal score > 1 were considered diarrheic. Fecal consistency was scored as follows: 0, normal; 1, pasty/semiliquid; 2, liquid; and 3, watery. ^†^Means with different letters (A and B) in the same column differ significantly (determined by Kruskal–Wallis rank sum test, *p* ≤ 0.05). ^@^AUC indicates diarrhea severity.

### Sample Collection and Processing

Rectal swabs were collected by inserting Dacron swab 3–5 cm into piglet’s rectum and rotating it against the rectal wall several times in the PRVC Cowden G1, RV0104, and RV0143 inoculated Gn pigs and mock pigs. Rectal swabs were processed by submerging the swabs into 2 mL of MEM-Gibco supplemented with 1% antibiotic-antimycotic (Anti-Anti; Life Technologies, Grand Island, NY, United States). Centrifugation was performed at 1,800 × *g* for 20 min at 4°C. Genomic RNA was extracted from rectal swab supernatants (50 μl) using MagMAX total RNA isolation kit (Life Technologies, Grand Island, NY, United States) according to the manufacturers protocol. RT-qPCR was performed using One-step RT-PCR Kit (Qiagen, Germantown, MD) using the following primer set and probe: RVC forward primer-5′ ATGTAGCATGATTCACGAATGGG 3′, RVC reverse primer-5′ ACATTTCATCCTCCTGGGGATC 3′, and Probe 5′-VIC-GCGTAGGGGCAAATGCGCATGA-TAMRA-3′. RT-qPCR conditions were as follows: reverse transcription at 50°C for 30 min, initial PCR activation at 95°C for 15 min, 40 amplification cycles with denaturation at 94°C for 1 min, annealing at 55°C for 1 min, extension at 72°C for 1 min, and final extension at 72°C for 10 min (Marthaler et al., 2014). PRVC shedding titers were calculated using the RT-PCR standard curve as described earlier.

### Histological Analysis of Small Intestinal Sections

Piglets from each group of 3-day-old (Cowden *n* = 2, RV0104 *n* = 2, RV0143 *n* = 2, and mock *n* = 1) and 3-week-old piglets (Cowden *n* = 3, RV0104 *n* = 3, RV0143 *n* = 4, and mock *n* = 2) were euthanized on PID 3. Sections (1 cm) of ileum, duodenum and jejunum were obtained and preserved in 10% formalin (Fisher Scientific, Hampton, NH, United States). Tissues were placed in 10% phosphate buffered formaldehyde (pH 7.0), dehydrated in graded alcohol, embedded in paraffin, and cut in 3-μm sections onto microscope slides, fixed, and stained with hematoxylin and eosin (H&E) then analyzed for histopathological changes. The images were captured using the imaging microscope Olympus B × 41 with camera Olympus DP72 and Olympus CellSens software at 10×.

### Detection of Cytokines in Serum and PRVC Antibodies by ELISA

Blood was collected at several time points: PID-5, PID2, PID7, and PID10, and serum was obtained by centrifuging at 2,095 × *g*. Serum samples were processed and analyzed for proinflammatory (TNF-α), innate (IFN-α), and Tregs (IL-10 and TGF-β), IL-17 and IL-22 cytokines as described previously with some modifications (Azevedo et al., 2006; Chattha et al., 2013). Briefly, Nunc Maxisorp 96-well plates were coated with anti-porcine TGF-β (1.5 μg/ml, clone 55B16F2), (Thermo Fisher Scientific, Waltham, MA, United States), or anti-porcine IFN-α (2.5 μg/ml, clone K9) (R&D systems, Minneapolis, MN, United States), overnight at 37°C for IFN-α or at 4°C for the other cytokines. Biotinylated anti-porcine IL-10 (1 μg/ml, clone 945A1A926C2), anti-porcine IFN-γ (0.5 μg/ml, clone A151D13C5), and anti-porcine TGF-β [0.4 μg/ml (Thermo Fisher Scientific, Waltham, MA, United States)], anti-porcine IFN-α (3.75 μg/ml, clone F17; R&D systems, Minneapolis, MN, United States), or anti-porcine TNF-α [0.4 μg/ml, goat polyclonal antibody (Ab), Kingfisher biotech, Saint Paul, MN, United States] were used for detection. Porcine IFN-α detection Ab was biotinylated using a commercial kit as described previously (Chattha et al., 2013). Plates were developed using 3-3′–5-5′ tetramethylbenzidine and cytokine concentrations were calculated as described previously (Azevedo et al., 2006). IgA PRVC geometric Ab mean titers (GMTs) in serum were measured by enzyme-linked immunosorbent assay (ELISA). To evaluate, serum PRVC-specific Ab levels, and cocktail virus-like-particles (VLPs) (100 ng) consisting of VP4, VP2, VP6, and VP7 of RV0104, Cowden or RV0143 were used to coat Nunc Maxisorp 96-well plates overnight at 4°C. Plates were washed 5× using PBS pH 7.4 with 0.05% tween (Life Technologies, Grand Island, NY, United States; Chepngeno et al., 2019). Plates were blocked using 100 μL of 1% bovine serum albumin diluted in PBS pH 7.4 with 0.05% tween for 2 h at room temperature and washed 5× using PBS pH 7.4 with 0.05% tween. Serum samples (50 μL) were diluted (1:4, 1:16, 1:64, and 1:256), added and incubated for 2 h at room temperature and washed 5× using PBS pH 7.4 with 0.05% tween. Horseradish peroxidase linked mouse anti-pig IgA (100 μL,1:5000) (Bio-Rad, Hercules, CA, United States) was added and incubated 1 h at room temperature, then washed 5× using PBS pH 7.4 with 0.05% tween. 3, 3′, 5, 5′-Tetramethylbenzidine substrate A and B were mixed (1:1) and 100 μL were added to each well for 3 min. The reaction was stopped using 100 μL of 9.8% phosphoric acid. Absorbance was recorded at 450 nm using Spectra Max 340PC384 microplate reader. The ELISA PRVC Ab titer was expressed as the reciprocal of the highest dilution that had a corrected *A*450 value [sample absorbance in the PRVC positive control serum samples (serum collected from Gn piglets inoculated with PRVC) wells minus sample absorbance in PRVC negative serum (serum collected from Gn piglets with no virus) greater than the cut-off mean corrected *A*450 value of negative controls plus 3 standard deviations].

### Statistical Analysis

Differences in the mean levels of serum cytokines and PRVC IgA Ab titers were analyzed by Mann-Whitney *U* test. Statistical differences in diarrhea scores and PRVC RNA shedding titers were done by Kruskal–Wallis rank sum test. All statistical analyses were performed using GraphPad Prism version 7 (GraphPad software, Inc., La Jolla, CA, United States). *P* values < 0.05 were considered statistically significant for all comparisons.

## Results

### Comparative Sequence Analysis

Analysis of complete sequences (with the exception of the RV0104 VP4 gene, for which almost complete ∼98% sequence was obtained) of the 11 genomic segments of RV0104 and RV0143 strains confirmed that the new strains RV0104 and RV0143 are only distantly related to one another and to the historic Cowden strain. For different genes, nucleotide identity between RV0104 and RV0143 ranged from 73% (NSP1) to 97% (NSP2) with overall higher nucleotide identity between RV0104 and Cowden noted in VP1 an NSP (Table 2). However, higher overall nucleotide identity (especially in most non-structural protein genes) was observed between RV0143 and Cowden. Specifically, VP1, VP2, VP6, NSP1, NSP2, NSP3, and NSP5 genes were more similar between Cowden and RV0143 (85–94%), whereas VP3, VP4, VP7, and NSP4 shared higher nucleotide identity between Cowden and RV0104 (78–94%; Table 2).

**TABLE 2:** Nucleotide identity between RVC/Pig-wt/USA/RV0104/2011/G3P18, RVC/Pig-wt/USA/RV0143/2012/G6P5, RVC Cowden (G1P1) strain and other RVCs of human, bovine and porcine origin.

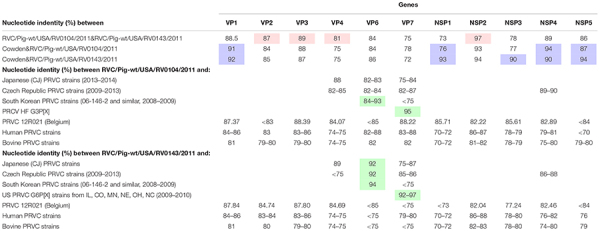

*Light pink shading was used to emphasize highest nucleotide identity between RV0104 and RV0143 strains; light blue – between RV0104 and/or Light pink shading was used to emphasize highest nucleotide identity between RV0104 and RV0143 strains; light blue – between RV0104 and/or RV0143 and Cowden, and light green – between RV0104/RV0143 and other PRVC strains.*

VP4 gene: A nucleotide identity of 81% was observed between the RV0104 (identified as P7 genotype) and RV0143 (P5 type) VP4 genes. At the same time, their VP4 genes had lower similarity (75%) to that of Cowden strain (Table 2). Of interest, both strains shared >84% nucleotide identity with a recent Belgian PRVC strain 12R021 characterized as P5 genotype.

VP7 gene: The complete nucleotide sequences of the VP7 gene of the RV0104 (G3) and RV0143 (G6) strains were compared with complete VP7 gene sequences of other RVC strains available in the GenBank database using BLASTn search. Sequence comparison indicated that RV0143 strain was most closely related to PRVC G6 strains from the US (IL, CO, NE, OH, and NC) from 2009–2010 with sequence identities for VP7 gene ranging from 92–97%; while VP7 gene of the other strain (RV0104) was most closely related to prototype RVC/Pig-wt/USA/HF/xxxx/G3Px strain with sequence identity of 95%. The two strains were distantly related to one another with sequence identity of only 75% and they were also distantly related to Cowden (78% and 72%; Table 2) and to other PRVC G-types, human and bovine strains with sequence identities ranging from 72 to 88%. RV0104 shared the highest nucleotide identity with the recently characterized Belgian PRVC strain 12R021 of 88.2% also identified as G3 genotype (Table 2).

VP6 gene: Only, 84% and 86% nucleotide identity were shared between Cowden and RV0104 and RV0143 VP6 genes. Full length sequences of the VP6 gene of the RV0104 and RV0143 strains showed ∼84% nucleotide identity to each other and 84–94% nucleotide identity to other porcine strains. RV0104 was most closely related to some PRVC strains from South Korea (from 2008–2009), sharing nucleotide identity of up to 93% in theVP6 gene. RV0143 VP6 also shared highest the identity of 94% with the latter strains, followed by recent Japanese and Czech PRVC strains sharing nucleotide identity of 92% (Table 2).

VP1, VP2, and VP3 genes: Higher nucleotide identity was noted between VP1 gene of RV0104 and RV0143 strains and Cowden VP1, while VP2, and VP3 genes of these three strains shared lower-moderate nucleotide identity (84–89%).

Among all genes for non-structural proteins, NSP2 genes of RV0104 and RV0143 strains shared the highest nucleotide identity with one another (97%). They also were highly genetically related to the NSP2 gene of Cowden strain sharing 93–94% of nucleotide identity. The sequence analysis of the full length NSP4 gene showed that the two field strains (RV0104 and RV0143) shared 89% nucleotide identity. They were more closely related to prototype Cowden strain with sequence identity of 94% and 90% for RV0104 and RV0143, respectively (Table 2), while distantly related to human (76–82%) and bovine (74–80%) strains. Of interest (except for NSP3) the NSP genes of Cowden and RV0143 shared higher nucleotide identity with one another than with RV0104. Overall, our molecular analysis suggests that PRVC strains are likely to evolve via continuous mutation and reassortment events as observed for PRVA strains.

### PRVC Diarrhea and Virus Shedding Titers

**FIGURE 1:**
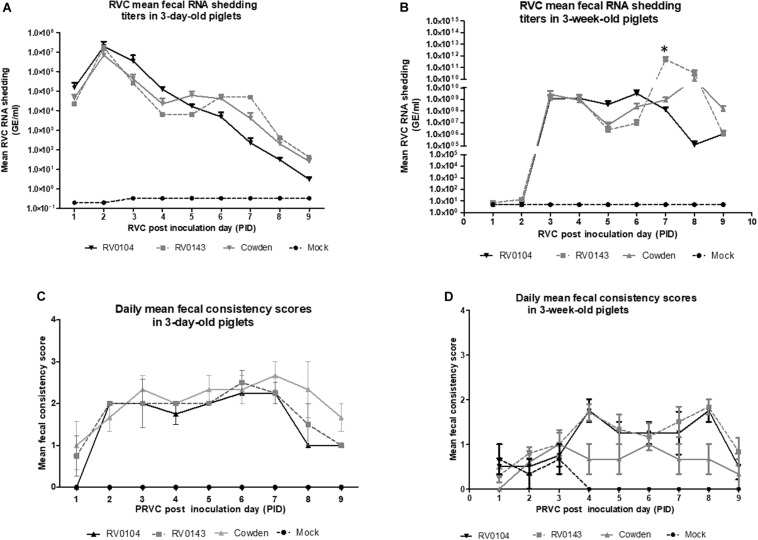
Mean (± SEM) daily fecal diarrhea score and mean (± SEM) PRVC diarrhea fecal shedding of porcine RVC infected Gn piglets. 3-day-old and 3-week old Gn piglets were infected with 10^5^ RNA copy/ml of porcine PRVC Cowden, RV0104, and RV0143 and mock and piglets were examined daily for **(A,B)**. fecal RNA shedding (RNA copy/ml) and **(C,D)**. diarrhea and their fecal score noted as follows: 0 – normal = solid; 1- pasty; 2- semi-liquid; and 3, liquid diarrhea from PID 1-PID 9 and mean (± SEM) daily fecal diarrhea was calculated from the scores.

All 3-day-old infected piglets developed severe diarrhea and occasional lethargy, although the median cumulative diarrhea score for RV0104 infected piglets was significantly lower when compared with Cowden and RV0143 infected piglets (Table 1 and Figure 1A). Peak virus shedding mean titers were numerically higher in RV0104 and RV0143 when compared with Cowden in 3-day-old piglets. However, no differences were noted in shedding onset or duration (Figure 1 and Table 1).

Three-week-old RV0104 and RV0143 piglets also developed diarrhea; however, as expected, the onset of diarrhea and virus shedding was delayed and lasted a shorter period compared with 3-day-old infected piglets (Table 1 and Figures 1C,D). Three-week-old Cowden infected piglets did not develop diarrhea, as reflected in significantly lower median cumulative diarrhea scores; however, these piglets actively shed virus post infection. Finally, peak shedding mean titers and mean shedding and diarrhea duration were significantly higher in RV0143 infected piglets compared with Cowden and RV0104 infected 3-week-old piglets coinciding with an earlier onset of RVC RNA shedding in RV0143 piglets.

### PRVC Cowden Strain Induced the Highest Levels of IL-22 Cytokine in 3-Day-Old Gn Pigs

**FIGURE 2:**
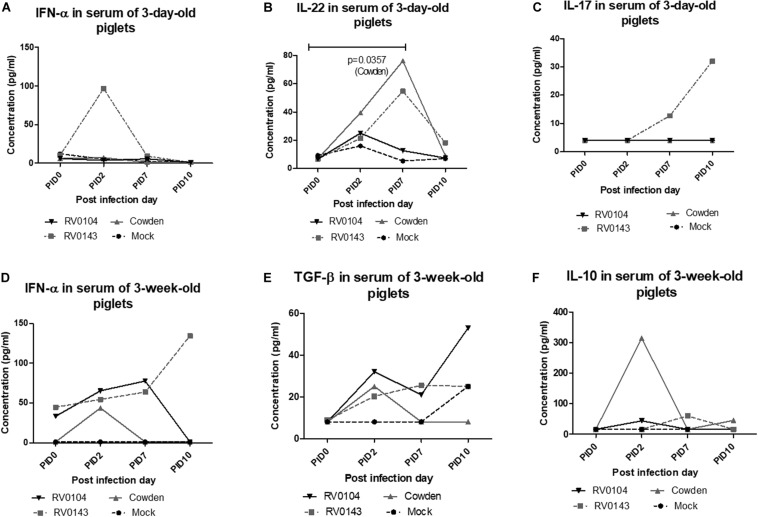
Mean (± SEM) concentrations of innate, proinflammatory, Th1, and T regulatory cytokines detected in serum of 3-day-old Gn piglets inoculated with 10^5^ RNA copy/ml of porcine RVC Cowden (*n* = 5), RV0104 (*n* = 7), and RV0143 (*n* = 7) and mock (*n* = 4) (**A**. IFN-α; **B**. IL-22, and **C**. IL-17) and 3-week-old Gn piglets inoculated with 10^5^ RNA copy/ml of porcine RVC Cowden (*n* = 5), RV0104 (*n* = 7), and RV0143 (*n* = 7) and mock (*n* = 3) (**D**. IFN-α; **E**. TGF-β and **F**. IL-10). Serum was collected once before and three times after PRVC inoculation (PID2, PID7, and PID10).

We compared the serum levels of innate (IFN-α), proinflammatory (IL-4, IL-6, and TNF-α), Th1 (IFN-γ and IL-12), Treg (IL-10 and TGF-β), and Th17 (IL-17 and IL-22) cytokines in piglet’s serum. In 3-day-old infected piglets, IFN-α and IL-17 were highest in the RV0143 Gn infected piglets peaking at different respective PIDs (Figures 2A–C). Serum IL-22 concentrations in Cowden and RV0143 3-day-old infected Gn piglets were significantly (*P* = 0.0357) and numerically higher, respectively, compared with mock Gn piglets (Figure 2B). We did not observe any statistically significant differences in the levels of IL-4, IL-6, TNF-α, IFN-γ, IL-12, IL-10, and TGF-β in serum samples of the 3-day-old piglets (Data not shown). In 3-week-old piglets, IL-10 was numerically higher in Cowden inoculated piglets at PID2 when compared with RV0104 and RV0143 inoculated piglets, while IFN- α increased ∼3× in RV0143 inoculated piglets at PID10. In 3-week-old piglets, TGF- β levels increased and peaked at PID10 in all PRVC inoculated compared with mock Gn piglets (Figure 2E). No trends were evident for IL-4, IL-6, TNF-α, IFN-γ, IL-12, IL-17, and IL-22 cytokines in serum samples of the 3-week-old piglets (Data not shown).

### Cowden Inoculated Pigs Had Higher PRVC-Specific IgA Responses

**FIGURE 3:**
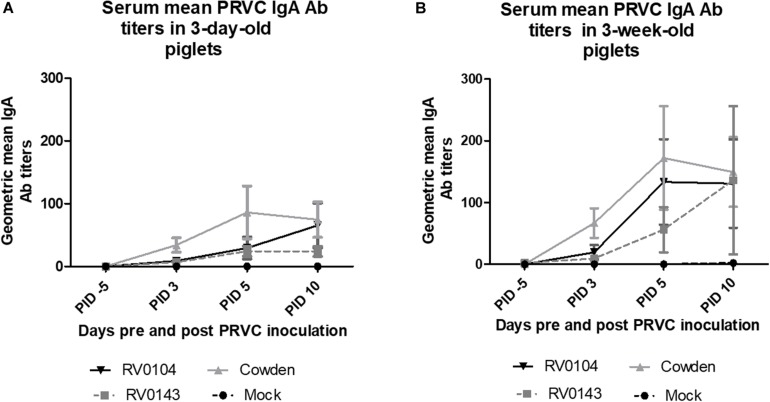
PRVC serum geometric mean IgA antibody titers. **(A)** 3-day-old Gn piglets were inoculated with 10^5^ RNA copy/ml of porcine PRVC Cowden (*n* = 5), RV0104 (*n* = 7), RV0143, (*n* = 7) or mock (*n* = 4). **(B)** 3-week-old Gn piglets were inoculated with 10^5^ RNA copy/ml of porcine RVC Cowden (*n* = 5), RV0104 (*n* = 7), RV0143 (*n* = 7) or mock (*n* = 3). Serum was collected 5 days pre-inoculation (-5PID) and three times post PRVC inoculation (3PID, 5PID, and 10 PID).

In contrast to lower PRVC diarrhea and shedding, both 3-day-old and 3-week-old Cowden challenged piglets had higher titers of PRVC IgA; whereas RV0143 induced lowest levels of mean IgA of the three strains in both 3-day-old and 3-week-old piglets (Figures 3A,B). We observed 2-fold higher PRVC IgA geometric mean Ab titers in inoculated 3-week- old piglets when compared with 3-day-old piglets, consistent with higher maturity of the immune system in older piglets.

### Histopathological Examination

**FIGURE 4:**
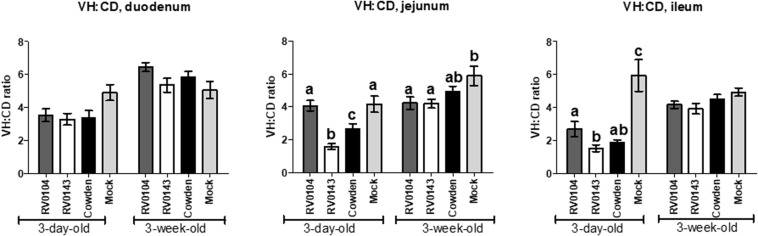
Mean (± SEM) ratios of villi height and crypts depth of duodenum, jejunum and ileum sections of PRVC inoculated gnotobiotic piglets (3-days-old and 3-week-old) at PID 3. The tissue sections were preserved in 10% buffered formalin before histopathology. The ratio of villi height and crypt depths was used to determine the villous atrophy was follows; normal (>4.0:1), mild atrophy (3.1–4.0:1), moderate atrophy (2.1–3.0:1), and marked atrophy (1.1–2.0:1). Different letters (a,b,c) represent statistical significance within the group.

**FIGURE 5:**
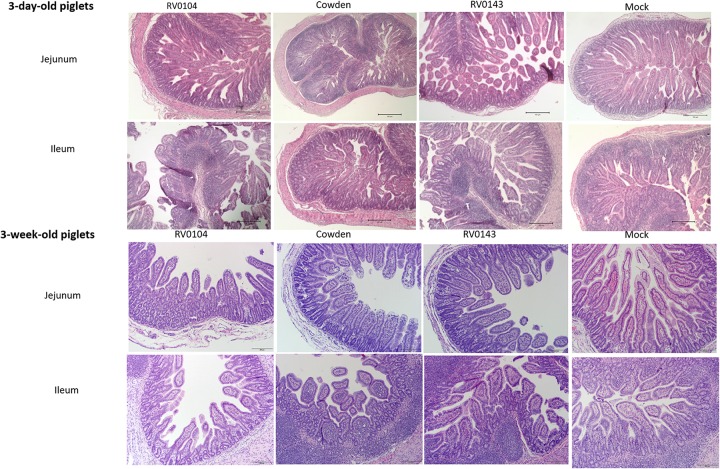
Villous atrophy and structural changes in jejunum and ileum of PRVC (Cowden, RV0104, and RV014) inoculated piglets at PID3. Tissues were preserved in 10% formalin before Hematoxylin and eosin staining. The images were captured using the imaging microscope Olympus B × 41 with camera Olympus DP72 and Olympus CellSens software at 10×.

Minimal to mild villous atrophy [villi height to crypt depth (VH: CD) ratios] was observed in duodenum of all 3-day-old inoculated piglets (Figure 4A). 3-day-old PRVC Cowden and RV0104 strains inoculated piglets showed mild-moderate and moderate-marked villous atrophy (Figures 4B,C), mild submucosal edema and mild multifocal lymphohistiocytic enteritis in jejunum and ileum sections (Figure 5). Mild multifocal to coalescing villous enterocytes vacuolar degeneration and mild submucosal edema were noted in most piglets in these groups. Mild crypt hyperplasia was also confirmed in one RV0104 inoculated 3 day-old-piglet. In contrast, marked villous atrophy was noted in jejunum and ileum of RV0143 inoculated piglets (Figures 4B,C), with no intact villi remaining in those sections of one piglet. Mild segmental submucosal edema and marked diffuse epithelial vacuolar degeneration were also observed in jejunum and ileum of RV0143 inoculated piglets, respectively. Thus, villous atrophy was significantly more severe in jejunum of RV0143 inoculated 3-day-old piglets than in jejunum of those inoculated with RV0104 and Cowden, and significantly or numerically more severe in ileum of RV0143 inoculated 3-day-old piglets than in ileum of those inoculated with RV0104 or Cowden, respectively.

In 3-week-old inoculated piglets, mild villous atrophy was observed in jejunum and ileum (Figures 4A–C, 5). There were no significant differences observed in jejunum, duodenum and ileum of these piglets compared with mock; however, the ratios of villi heights to crypt depths (VH:CD) were numerically lower in jejunum of RV0104 and RV0143 when compared with mock.

## Discussion

PRVC has been increasingly detected in suckling neonatal piglets causing diarrhea and leading to significant economic losses to farmers and the pork industry (Morin et al., 1990; Martella et al., 2007; Amimo et al., 2013; Marthaler et al., 2014; Moutelikova et al., 2014). It is not well understood if the increase in prevalence of PRVC in neonatal nursing piglets is associated with lack of herd immunity or altered pathogenicity of the new vs. historic PRVC strains. Susceptibility to RV clinical disease have been shown to be age related (Conner and Ramig, 1997), and the difference in age specificity is not well understood and is speculated to be unrelated to receptors (Ramig, 2004; Pott et al., 2012).

As expected, all three strains induced severe diarrhea in 3-day-old Gn piglets; the mean fecal shedding titers in the RV0104 and RV0143 3-day-old inoculated piglets were twice as high on PID 2 when compared with Cowden G1 inoculated piglets. Cowden inoculated 3-week-old piglets did not develop diarrhea, although they shed virus. In contrast to the 3-week-old Gn pigs inoculated with Cowden G1, RV0104 and RV0143 inoculated 3-week-old pigs developed PRVC diarrhea; although diarrhea and shedding were decreased compared with those in the 3-day-old inoculated piglets. These observations suggest that the new PRVC strains evaluated in this study can be more pathogenic and possess an enhanced ability to spread in swine herd.

During acute infection, villi structural changes were observed in ileum and jejunum of both 3-day-old and 3-week-old inoculated piglets with less noticeable changes in duodenum. This result is in agreement with a previous PRVC pathogenesis study where villous atrophy was observed in Gn piglets inoculated with PRVC Cowden strain with less destruction observed in duodenum as RV replication is believed to be dominant in the jejunum and ileum (Bohl et al., 1982). The more substantial destruction of small intestinal villi in 3-day-old piglets may be due to an underdeveloped immune system and/or lower turnover rate of enterocytes in neonatal piglets (Azevedo et al., 2013). The increased severity of pathological changes in the intestinal sections of the RV0143 inoculated 3-day- and 3-week-old pigs is consistent with the increased fecal virus shedding in these piglets. The overall severe diarrhea (highest score = 3) in the 3-day-old piglets did not allow us to make a distinction between the three different strains. However, 3-week-old piglets inoculated with the new PRVC strains developed diarrhea in contrast to those inoculated with Cowden, which supports our other findings suggestive of increased pathogenicity of the current PRVC strains.

In contrast to the decreased intestinal pathology and PRVC shedding, PRVC IgA Ab titers were highest in Cowden inoculated pigs (3-day- and 3-week-old). While RV0143 inoculated piglets that shed the highest PRVC RNA amounts had the lowest PRVC IgA Ab titers. This may be a result of increased immune evasive potential since NSP1 of RV has been shown to degrade interferon factor 3 resulting in evasion of innate immunity which in turn might affect IgA Ab production (Barro and Patton, 2005).

Rotaviruse infection elicits innate immune responses in the small intestinal epithelial cells, inducing proinflammatory signaling and releasing type I and II IFNs and other cytokines involved in antiviral immunity (Holloway and Coulson, 2013). IL-22 that belongs to the IL-10 family has recently been found to play a critical protective role against viral infections in the mucosal surfaces (Ouyang and Valdez, 2008; Sonnenberg et al., 2011). IL-22 was associated with the expression of antimicrobial defensin proteins and the promotion of tissue barrier in intestinal epithelial cells, protecting against mucosal viral infections (Xue et al., 2017). We observed that IL-22 was significantly (*P* = 0.0357) or numerically higher in 3-day-old Gn piglets inoculated with PRVC Cowden or RV0143 but not in the RV0104 or mock Gn piglets. Significantly higher of IL-22 when compared with mock could have contributed to the decreased virus shedding titers observed at peak (PID 2) in Cowden inoculated compared with the RV0104 and RV0143 3-day-old inoculated Gn piglets. IL-22 production and functions are influenced by different cytokines including IL-17, IFN-α, IFN-γ, or TNF-α (Liang et al., 2006; Eyerich et al., 2009; Guilloteau et al., 2010; Sonnenberg et al., 2010), most of which were up-regulated in the RV0143 inoculated piglets at variable time-points. Surprisingly, none of those cytokine levels were increased in the Cowden inoculated piglets that had the highest levels of serum IL-22, while all the cytokine levels were intermediate in the RV0104 3-day-old piglets. It is noteworthy that upregulation of IL-22 production alone (seen in Cowden G1 inoculated piglets) may contribute to increased proliferation and survival of intestinal epithelial cells, decreasing virus shedding and alleviating intestinal pathology in neonatal piglets. In contrast, upregulation of IL-22 production in combination with IL-17 (seen in RV0143 piglets) could have enhanced pro-inflammatory signaling, aggravating intestinal pathology (Parks et al., 2015). IL-10 was higher at PID2 in 3-week-old piglets inoculated with Cowden strain which might have reduced pro-inflammatory mediated diarrhea in these piglets. This coincided with increased PRVC IgA Ab titers in those piglets, which is consistent with the fact that IL-10 promotes B cell proliferation, survival, and differentiation into plasma cells (Rousset et al., 1992). Jiang et al reviewed data from a variety of RV studies in humans which demonstrated that when RV-specific IgA antibodies are present in serum at elevated levels, they correlate with intestinal sIgA (Jiang et al., 2002).

Another, interesting observation is that PRVC RV0143 strain was most potent (and RV0104 was intermediate) at up-regulating IFN-α responses in piglets of both ages. Additionally, both current PRVC strains induced prolonged elevation of IFN-α levels in the 3-week-old piglets, in contrast to Cowden strain, in which levels that briefly peaked at PID2 and then declined. Prolonged up-regulation of IFN-α responses is consistent with more pro-inflammatory environment and can decrease the anti-inflammatory activity of IL-10 (Rousset et al., 1992). These findings emphasize once again that the pathogenesis of these three PRVC strains in a Gn piglet model differs. TGF-β peaked at PID10 in all infected piglets. This observation is expected as TGF-β has been shown to control inflammation during infection and tissue repair after injury (Li et al., 2006).

While our sequence analysis did not identify a single gene that could be responsible for the observed increased pathogenicity of at least one new PRVC strain (RV0143), we speculate that it could have resulted from acquisition of genetically distinct (from Cowden) VP4 and VP7 genes from other known and unknown PRVC strains by a parental RVC strain sharing high similarity with Cowden in its non-structural genes.

## Conclusion

In conclusion, we have shown that the newly identified dominant strains RV0104 and RV0143 induce clinical disease in neonatal Gn piglets with relatively higher mean PRVC fecal RNA shedding titers compared to the historic Cowden strain. In addition, we have also documented that the RV0143 strain caused increased destruction of villi in jejunum and ileum sections of the small intestine compared with the other two strains. Furthermore, we demonstrated that Cowden induced higher mean levels of IL-22 and serum PRVC IgA Ab titers which could have contributed to the decreased virus shedding and intestinal pathology.

Lastly, we have shown that 3-week-old piglets inoculated with new PRVC strains developed clinical disease, in contrast to those inoculated with Cowden, and shed viral RNA at higher titers, suggesting that they might contribute to enhanced spread and persistence of PRVC. Finally, the sharp contrast between the increased replication/pathogenicity and diminished PRVC IgA Ab responses associated with the current strains (especially RV0143) may indicate that they have evolved some immune evasion mechanisms. Collectively, these differences in the pathogenesis of the new, genetically distinct PRVC strains (RV0104 and RV0143) may contribute to the increased prevalence of PRVC infection and diarrheal disease observed in neonatal suckling piglets.

## Data Availability Statement

The datasets generated for this study can be found in the NCBI gene bank- MN809633 (VP1), MN809634 (VP2), MN809635 (VP3), MN809636 (VP4), MN809637 (VP6), MN809638 (VP7), MN809639 (NSP1), MN809640 (NSP2), MN809641 (NSP3), MN809642 (NSP4), MN809643 (NSP5), MN809644 (VP1), MN809645 (VP2), MN809646 (VP3), MN815932 (VP4), MN809647 (VP6), MN809648 (VP7), MN809649 (NSP1), MN809650 (NSP2), MN809651 (NSP3), KC164673 (NSP4), MN809652 (NSP5), M74216 (VP1), FJ970917 (VP2), M74219 (VP3), M74218 (VP4), M94157 (VP6), M61101 (VP7), X60546 (NSP1), X65939 (NSP2), M69115 (NSP3), AF093202 (NSP4), and X65938 (NSP5).

## Ethics Statement

All animal experiments were approved by the Institutional Animal Care and Use Committee at The Ohio State University, prior to experimentation (2009A0146). All of the pigs were maintained, sampled, and euthanized according to guideline of public health service policy on humane care and use of lab animal and USDA animal awareness act guideline for animal care and use of lab animal (Public Health Service, 2002 and US Department of Agriculture, 1985). Pigs were euthanized using Telazol-Ketamine-Xylazine intra-muscular injection for anesthesia which was followed by electrocution.

## Author Contributions

AV and LS conceived and designed the experiments. JC, ST, AD, FP, CB, MH, MR, JH, and DM conducted experiments. JC, CB, ST, and AV analyzed data. JC and AV wrote the manuscript. JC, AV, and LS edited the manuscript. All authors critically read and approved the manuscript for publication.

## Conflict of Interest

The authors declare that the research was conducted in the absence of any commercial or financial relationships that could be construed as a potential conflict of interest.

## Supplementary Material

TABLE S1 Summary of gene specific primers used for RVC detection and sequencing.
