# Supplementary material for: Comparative Sequence Analysis of Historic and Current Porcine Rotavirus C Strains and Their Pathogenesis in 3-Day-Old and 3-Week-Old Piglets
Source: Front Microbiol. 2020 Apr 24;11:780. doi: 10.3389/fmicb.2020.00780 (PMC7197332; doi:10.3389/fmicb.2020.00780)
Supplement: TABLE S1 — Summary of gene specific primers used for RVC detection and sequencing. [file Data_Sheet_1.PDF]

Primers	Name	Sequence	Segment	Amplicon size(bp)
Diagnostic (Partial VP6)	VP6F<br>VP6R	ACAGTATTTCAGCCAGGDTTTC<br>AGCCACATAGTTCACATTTCATC	1095-1116	260
Amplification and sequencing	RV0104VP41F<br>RV0104VP41R<br>RV0104VP42F<br>RV0104VP42R	ATGGCGTCCTCACTTTA<br>AAGCTGGTCTCCTCACTGATTAAAT<br>GATTATTGGGACGATTCAG<br>AAGCTGGTCTCCTCACTGATTAAAT	1-17<br>1192-1211<br>1075-1092<br>2208-2232	 1211<br><br>1157
